# Intra-amniotic *Candida albicans* Infection Treated With Liposomal Amphotericin B With a Successful Neonatal Outcome

**DOI:** 10.1093/ofid/ofae047

**Published:** 2024-01-31

**Authors:** Norma Urbano Gutiérrez, María José Vergara López, Camila Álvarez Bustos, Cristian Contreras Vidal, Jorge A Carvajal, Nicolás Severino, Ady Giordano, Soledad Urzúa Baquedano, Teo Feuerhake, Ricardo Rabagliati, María Elvira Balcells

**Affiliations:** Departamento de Obstetricia, Escuela de Medicina, Pontificia Universidad Católica de Chile, Santiago, Chile; Departamento de Obstetricia, Escuela de Medicina, Pontificia Universidad Católica de Chile, Santiago, Chile; Departamento de Obstetricia, Escuela de Medicina, Pontificia Universidad Católica de Chile, Santiago, Chile; Departamento de Obstetricia, Escuela de Medicina, Pontificia Universidad Católica de Chile, Santiago, Chile; Departamento de Obstetricia, Escuela de Medicina, Pontificia Universidad Católica de Chile, Santiago, Chile; Programa de Farmacología y Toxicología, Escuela de Medicina, Pontificia Universidad Católica de Chile, Santiago, Chile; Departamento de Medicina Intensiva, Escuela de Medicina, Pontificia Universidad Católica de Chile, Santiago, Chile; Escuela de Química, Facultad de Química y de Farmacia, Pontificia Universidad Católica de Chile, Santiago, Chile; Departamento de Neonatología, Escuela de Medicina, Pontificia Universidad Católica de Chile, Santiago, Chile; Departamento de Anatomía Patológica, Escuela de Medicina, Pontificia Universidad Católica de Chile, Santiago, Chile; Departamento de Enfermedades Infecciosas del Adulto, Escuela de Medicina, Pontificia Universidad Católica de Chile, Santiago, Chile; Departamento de Enfermedades Infecciosas del Adulto, Escuela de Medicina, Pontificia Universidad Católica de Chile, Santiago, Chile

**Keywords:** *Candida albicans*, cervical insufficiency, chorioamnionitis, fungal chorioamnionitis, intra-amniotic infection, liposomal amphotericin B

## Abstract

Intra-amniotic infection with *Candida* species is an uncommon but severe condition with high fetal morbimortality and no established clinical guidelines for its management. We report a *Candida albicans* intra-amniotic infection diagnosed in a 25-week pregnant woman, successfully treated with high-dose liposomal amphotericin B. Pregnancy was prolonged until 30 weeks, and despite persistently positive *Candida* cultures in amniotic fluid, a healthy newborn was delivered without evidence of systemic infection. Amphotericin concentration was determined at birth, revealing levels over 30 times higher in mother's and cord blood than in the amniotic fluid, probably explaining the clinical protection despite failure in obtaining fungal clearance.

Intra-amniotic infection (IAI), also referred as chorioamnionitis, is characterized by the presence of microorganisms in the amniotic cavity, leading to an inflammatory process that can affect the placenta, fetal membranes, amniotic fluid, or the fetus itself [1–3]. It can be suspected by clinical symptoms and signs (clinical chorioamnionitis) or confirmed by histologic evidence of placental infection/inflammation or by a positive amniotic fluid test result [1, 3, 4]. Intra-amniotic infection is typically polymicrobial, secondary to genital mycoplasmas, aerobic and anaerobic vaginal bacteria, or enteric microbiota that ascend into the amniotic cavity. Less common routes of infection are hematogenous dissemination and accidental inoculation from invasive procedures [3, 5]. The reported prevalence for IAI is 2%–5% of all pregnancies, being more frequent in preterm deliveries [3, 4, 6]. Intra-amniotic infections can lead to acute maternal complications such as maternal sepsis, postpartum hemorrhage, puerperal endometritis, and respiratory distress syndrome [1]. In the newborn, IAI causes acute infection with severe morbidity and mortality and with potential for short- and long-term sequelae, including pulmonary and neurodevelopment disorders such as cerebral palsy [3].

Fungal IAIs are uncommon, despite *Candida* vulvovaginitis being common in pregnancy (18%–25%) [7–9]. The risk of ascending infection ranges from 0.8% to 2% [7, 8], with a reported prevalence of *Candida* IAI in 0.3% of all deliveries [10]. Described risk factors are prelabor membrane rupture, the presence of foreign bodies such as intrauterine devices (IUDs) or cerclages, and previous in vitro fertilization [8, 10–13]. The main causal agent is *Candida albicans*, although other species such as *C. glabrata*, *C. famata*, *C. parapsilosis*, and *C. kefyr* have been described [10]. Its clinical course and optimal management are not yet fully established, as most information relies on case reports and small case series [10]. *Candida* infection that develops in the first week of life in the newborn (“early congenital candidiasis”) has very high mortality—particularly at lower gestational ages and birth weights—reaching 71% in newborns <1500 g, with neurodevelopmental sequelae in up to 86% [8, 10].

We report the case of a pregnant woman with intra-amniotic *Candida albicans* infection treated with a long course of high-dose systemic liposomal amphotericin B, prolonging the latency period until delivery and achieving negative cultures in the newborn.

## CLINICAL CASE

**Figure 1.**
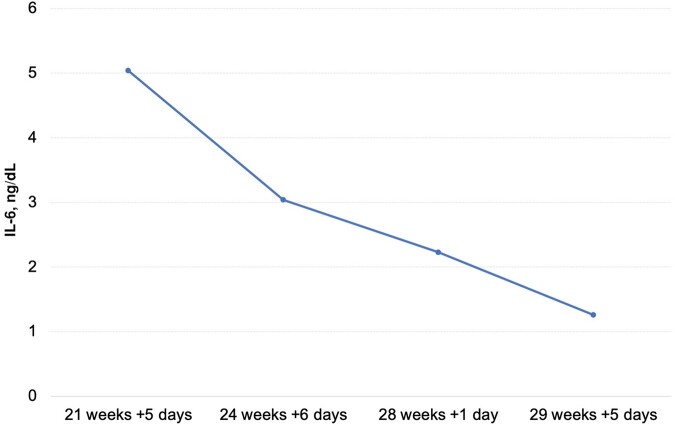
IL-6 concentration in amniotic fluid during antifungal treatment. Abbreviation: IL-6, interleukin-6.

A 29-year-old primigravid woman with a medical history of insulin resistance on metformin was referred to the obstetric unit of Hospital Clínico UC-CHRISTUS at 21 + 5 weeks gestation for the finding of cervical length of 2 mm during a routine ultrasound, with cervical funneling, without sludge. She was admitted in good condition with a uterus of normal tone and no contractions, and the speculoscopy revealed a 1-cm-dilated cervix with exposed membranes. Maternal blood inflammatory parameters were within normal range (white blood count 10.1 × 10^3^/µL, absolute neutrophil count 7.61 × 10^3^/µL, C-reactive protein 0.16 mg/dL). An amniocentesis was done; amniotic fluid (AF) analysis revealed a normal cell count but elevated interleukin-6 (IL-6), indicating intra-amniotic inflammation (Table 1, Figure 1). Blood, cervicovaginal, and AF cultures were obtained, and empirical antibiotic treatment was started with ceftriaxone, metronidazole, and clarithromycin. After 5 days of antibiotics, blood and AF bacterial cultures resulted negative, and *Candida albicans* was recovered from the cervicovaginal sample. A cervical cerclage was performed, and oral fluconazole was added as 2 doses of 150 mg each, separated by 7 days.

**Table 1.** Serial Amniotic Fluid Amniocentesis Laboratory Analysis Results

Gestational Age	Leukocyte Count^a^	Glucose^b^	LDH^c^	Gram Stain	Culture	IL-6^d^
21 wk +5 d	3 per field	35 mg/dL	122 U/L	No microorganism	Negative	5.04 ng/mL
24 wk +6 d	20 per field	34 mg/dL	165 U/L	Yeasts	*(+) Candida albicans*	3.04 ng/mL
28 wk +1 d	14 per field	36 mg/dL	127 U/L	Not performed	*(+) Candida albicans*	2.23 ng/mL
29 wk +5 d	6 per field	48 mg/dL	123 U/L	No microorganism	*(+) Candida albicans*	1.26 ng/mL

Abbreviations: IL-6, interleukin-6; LDH, lactate dehydrogenase.

^a^Normal amniotic fluid leukocyte count <50 leukocytes per field.

^b^Normal amniotic fluid glucose concentration <14 mg/dL.

^c^Normal amniotic fluid LDH concentration <400 U/L.

^d^Normal amniotic fluid IL-6 concentration <3 ng/mL.

After 21 days of antibiotic treatment (at 24 weeks + 6 days gestation), another amniocentesis was performed, finding persistently elevated IL-6 and yeasts on the gram stain. Betamethasone was administrated, and liposomal amphotericin B (L-AmB) at 4 mg/kg/d was indicated (Phosome B, Cipla Laboratory). Amniotic fluid culture confirmed the presence of *Candida albicans* with a minimal inhibitory concentration (MIC) of 0.25 for fluconazole and 0.5 for amphotericin B, both of which are within the susceptible range.

Strict maternal monitoring of inflammatory parameters, renal function, and plasma electrolytes ensued. Despite this, the patient developed hypokalemia (2.8 mEq/L), requiring frequent intravenous potassium and magnesium supplementation. Renal function remained unaffected. Serial maternal 1–3 β-D-glucan levels in plasma remained negative (<60 pg/mL), as well as blood cultures. The patient remained clinically asymptomatic, without signs of chorioamnionitis or of maternal candidemia, and her serum inflammatory parameters remained within normal range. The cervical cerclage was maintained, and fetal well-being and indemnity of the fetoplacental unit were evaluated weekly. Serial ultrasound confirmed adequate fetal growth, without anatomical malformations. Fetal neurosonography showed no alterations.

At 28 weeks + 1 day of gestational age, on day 21 of L-AmB treatment, a new amniocentesis showed normalization of IL-6 level, but a persistently positive *Candida albicans* culture in AF. Following a multidisciplinary team discussion, the L-AmB dose was increased (4.5 mg/kg/d) and maintained for 2 additional weeks, with the intention of delaying delivery.

At 29 + 5 weeks of gestation, on day 33 of L-AmB, the patient developed uterine contractions. The cerclage was removed, and a second course of corticosteroids was administered, maintaining the antifungal treatment. She progressed within hours into spontaneous labor, with cesarean delivery required due to failure in active phase progression. Amniocentesis was performed during labor, and AF analysis resulted noninflammatory but *Candida albicans* culture was still positive (Table 1, Figure 1).

A male newborn was delivered with birth weight 1706 g, birth length 40 cm, an Apgar score of 8 at 1 and 5 minutes, and a pH of 7.22 and base excess of −9.1 in cord gases analysis. He required supplementary oxygen briefly during immediate newborn care but no other oxygen requirements during the rest of his hospitalization. He was admitted to the neonatal intensive care unit for monitoring. Bacterial and fungal cultures (blood, urine, spinal fluid, skin) were taken, and deoxycholate amphotericin (D-AmB) was started. Echocardiogram, eye examination, and abdominal and brain ultrasound did not show evidence of *Candida* infection. Direct visualization of fungi in preauricular skin and perianal skin was also negative. After 5 days, with all cultures negative, amphotericin was stopped. He was discharged from the hospital healthy at 64 days of life.

The placenta was sent for pathological examination. Light microscopy showed focal and sparse neutrophilic infiltrates in the decidua capsularis, compatible with mild acute subchorionitis (maternal inflammatory response: early stage, grade 1) [14]. No infiltrates were present in the umbilical cord or the chorionic plate.

At birth, samples of maternal blood, newborn cord blood, and AF were sent for L-AmB level analysis to the laboratories at the Faculty of Chemistry and Pharmacy from Pontificia Universidad Católica de Chile. Total concentrations of L-AmB in the different matrices were analyzed by ultra performance liquid chromatography-mass spectrometry (UPLC MS/MS) [15, 16]. L-AmB concentrations were 75.7 mg/L in maternal blood, 54.8 mg/L in cord blood, and 2.4 mg/L in AF (Table 2; Supplementary Appendix).

**Table 2.** Liposomal Amphotericin B Concentration in Maternal Blood, Umbilical Cord Blood, and Amniotic Fluid at Birth

Sample	Concentrations ± SD, mg/L
Maternal blood	75.7 ± 5.45
Cord blood	54.8 ± 3.4
Amniotic fluid	2.4 ± 1.1

## DISCUSSION

We report a case of *Candida albicans* IAI treated with high-dose L-AmB that resulted in a healthy newborn without evidence of clinical or microbiologic infection. Given the infrequent occurrence of this condition and the absence of established standard recommendations, its management is typically guided by the clinical judgment of the treating physicians, for whom striking the right balance between maternal and fetal toxicities while considering clinical outcomes becomes a challenging task.

The treatment of invasive candidiasis requires the use of systemic antifungals. The Infectious Diseases Society of America Clinical Practice Guideline for the Management of Candidiasis recommends the use of amphotericin B as the first-line treatment for systemic candidiasis in pregnant women and the avoidance of azoles in the first trimester, but does not include a specific recommendation for IAI [17]. The pharmacokinetics of antifungal drugs in the human placenta and their actual concentration in the AF are only partially known [18]. Fluconazole, a systemic triazole with a fungistatic effect, crosses the placenta, reaching higher levels in fetal than in maternal blood, and is embryotoxic in animals, generating osteocartilaginous and craniofacial alterations [19, 20]. Its use is considered teratogenic in the first trimester, being D category in high doses (>400 mg/d) and C category in low doses (150 mg/d) as per the Food and Drug Administration classification, although treatment with a single dose of fluconazole in the first trimester (50–150 mg) has not demonstrated adverse effects in fetuses [21, 22]. Echinocandins, on the other hand, are inhibitors of the 1,3-1 beta-D-glucan synthetase, an enzyme that participates in the synthesis of the fungal wall, and they are fungicidal against most *Candida* species, but their use is also considered category C in pregnancy due to their embryotoxic potential [21].

Amphotericin B binds the ergosterol of the fungal membrane, leading to the formation of pores, ion leakage, and ultimately fungal cell death, a highly effective mechanism against most *Candida* species [21]. It has 4 different formulations: deoxycholate (D-AmB), liposomal (L-AmB), lipid complex, and colloidal dispersion, with varying degrees of toxicity. L-AmB formulations are significantly less nephrotoxic than D-AmB, allowing higher dose administration [23, 24]. In addition, total plasma concentrations of L-AmB are higher than those observed with D-AmB because the biologically active drug is released from the liposome only after direct contact with the fungus [25]. Amphotericin B is the safest systemic antifungal in pregnancy (category B) [21]. It crosses the placenta and achieves therapeutic concentrations in fetal circulation. Intravenous administration is the standard, but transcervical and transabdominal amnioinfusion administration have also been reported [26]. No teratogenic effects have been described in animals at doses 10 times higher than those used in humans [24]. Its pharmacodynamic and pharmacokinetic properties in the fetus are not fully characterized. According to reported data from conventional D-AmB use during pregnancy, its levels in cord blood and in the placenta remain within the MIC range for up to 4 weeks after the last dose. Research suggests that the drug accumulates in the tissues, which then act as a reservoir from which amphotericin B is slowly released back into circulation [27].

In the present case, we found that L-AmB AF levels were 30 times lower than in maternal blood. D-AmB concentrations in body fluids such as vitreous humor, pleural fluid, or normal amniotic fluid have been reported to be very low [18, 28]. Indeed, D-AmB levels in amniotic fluid were reported to be at least 6 times lower than in maternal blood in a case of disseminated maternal blastomycosis [29]. Whether the liposomal formulation of amphotericin further impairs the drug passage into AF is unknown, and there is a scarcity of published data on L-AmB pharmacokinetics in this maternal–fetal interphase. Additionally, the pharmacokinetics of L-AmB is more variable than that of D-AmB, a phenomenon that has been attributed to the saturation of the clearance mechanisms for liposomes containing amphotericin B, which can occur after consecutive infusions, especially at high doses [25].

Nonetheless, amphotericin B in both formulations achieves therapeutic concentrations in fetal circulation, with reports of cord blood vs maternal serum ratios ranging from 0.38 to 1 (0.72 in our case) and studies showing similar concentrations of D-AmB in umbilical cord, infant serum, and placental tissue [21, 27].

The prevalence of IAI caused by *Candida* species is not well established and varies among cases, being higher in preterm deliveries and preterm membrane rupture [8]. The accuracy in identifying the responsible microorganisms also plays a role, with a higher success rate when combining culture and polymerase chain reaction (PCR) techniques [30]. Nevertheless, *Candida* IAI is still an infrequent condition, as shown by various microbiological studies that found no *Candida* species in amniotic fluid in patients with rupture of membranes at term, 0.3%–1% positivity in patients with preterm labor and intact membranes, 4% positivity (by culture or PCR) in patients with clinical chorioamnionitis at term, and 5% positivity in patients with preterm prelabor rupture of membranes [30–34]. The clinical outcomes for *Candida* IAI have been reported in only a few cases and small series, generally with adverse outcomes [10]. A recent review of 123 chorioamnionitis cases in a tertiary medical center in Japan found a prevalence of 0.3% for *Candida* infections (diagnosed by isolation of *Candida* species in cultures from amniotic fluid, fetal/neonatal/placental, or histologic evidence of fungal infection). The most frequent predisposing condition was prelabor rupture of membranes, and the most common symptom was preterm labor. Cervical dilatation without uterine contraction was the clinical presentation in 8.9% of patients. Births between 22 and 36 weeks occurred in 60% of singleton pregnancies, with a mortality of 29%. Thirteen cases received antenatal treatment, and only 6 succeeded in having live infants. Fluconazole was the most commonly used antifungal, followed by amphotericin B, with variable routes of administration [10]. Furthermore, in a recent local case report of IAI by *Candida albicans* treated with fluconazole (400 mg intravenously daily), the patient evolved with rapid progression into preterm labor with delivery of a nonviable fetus with histologic evidence of fungal involvement of the umbilical cord [35]. On the other hand, the only published case of *Candida albicans* IAI also treated with systemic L-AmB reported a successful fungal clearance, although a secondary bacterial chorioamnionitis ensued [36].

In conclusion, *Candida* species IAI is an uncommon but severe complication of pregnancy that should be suspected in patients with IAI and initially negative bacterial cultures, particularly in the presence of risk factors. There is no consensus on first-line treatment, although amphotericin B is the only antifungal in the B category in pregnancy. Its liposomal formulation is well tolerated, and it has fewer adverse events, allowing higher doses. In this clinical case, treatment with L-AmB permitted a 1-month prolongation of pregnancy and resulted in a healthy newborn with no evidence of localized or systemic *Candida* infection despite failing to sterilize AF. Clinical research efforts should be oriented toward gathering better evidence for the treatment of these less common infections in pregnant women, and, as in this case, determination of L-AmB concentrations in both fetal and maternal samples can provide valuable insights for establishing optimal therapeutic levels in future treatment scenarios.

## Supplementary Material

ofae047_Supplementary_Data

## Supplementary Data

Supplementary materials are available at *Open Forum Infectious Diseases* online. Consisting of data provided by the authors to benefit the reader, the posted materials are not copyedited and are the sole responsibility of the authors, so questions or comments should be addressed to the corresponding author.
