# Supplementary material for: Intra-amniotic Candida albicans Infection Treated With Liposomal Amphotericin B With a Successful Neonatal Outcome
Source: Open Forum Infect Dis. 2024 Jan 31;11(2):ofae047. doi: 10.1093/ofid/ofae047 (PMC10873704; doi:10.1093/ofid/ofae047)
Supplement: ofae047_Supplementary_Data [file ofae047_supplementary_data.docx]

**Supplementary Appendix**

Method for Amphotericin B levels measurement:

The total concentrations of L-AmB in the different matrices were analyzed by ultra-high performance liquid chromatography- mass spectrometry (UHPLC-MS/MS) with a Sciex triple Quad 4500 with Turbo V electrospray, coupled to an Eksigent Ekspert Ultra LC100 system (AB/Sciex Concord, Ontario, Canada). The chromatographic separation was carried out on a GL Sciencie Inersil C8 column (100 mm x 4.6 mm id, 3µm) at controlled temperature (40ºC) with mobile phase consisting of acetonitrile: 0.1% formic acid in water 20:80 at a flow rate of 0,5 mL/min. The instrument was operated using electrospray ionization in positive ion mode and the operating parameters were optimized to obtain optimum performance. The MS/MS transitions 924>906 and 924>743 for Amphotericin B were used. Vancomycin was used as internal standard with MS/MS transition of 725>144 and 725>100. Calibration curves were prepared by spiking stock solution of L-AmpB on blank plasma and ranged from 0,5 to 10 mg/L. Specificity was demonstrated by comparing chromatograms of blank plasma before and after enrichment with L-Amb. Accuracy and precision were determined using results from three replicates of the low and high control samples, assayed on three days for intra- and inter-day assays. Matrix effect was evaluated by analyzing 3 replicates of low and high control samples added with blank plasma. Limit of quantification (0,05 mg/L) was evaluated by signal-to-noise ratio. Sample preparation involves protein precipitation; briefly 200 uL of samples was mixed with 200 uL SI solution and 1,4 mL methanol. After vortexing, the mixture was centrifugated at 8000 rpm for 8 min. The supernatant was evaporated to dryness and reconstituted on 200 uL of acetonitrile (15,16).
